# Biosynthesis of Iron Oxide Nanoparticles by Marine *Streptomyces* sp. SMGL39 with Antibiofilm Activity: In Vitro and In Silico Study

**DOI:** 10.3390/molecules29194784

**Published:** 2024-10-09

**Authors:** Sara A. Attea, Mosad A. Ghareeb, Ayda K. Kelany, Heba K. A. Elhakim, Khaled S. Allemailem, Sarah I. Bukhari, Fatma B. Rashidi, Ahmed A. Hamed

**Affiliations:** 1 Biochemistry Division, Chemistry Department, Faculty of Science, Cairo University, Giza 12613, Egypt; saraawad@gstd.sci.cu.edu.eg (S.A.A.); drheba_76@yahoo.com (H.K.A.E.); fabdallah@sci.cu.edu.eg (F.B.R.); 2 Medicinal Chemistry Department, Theodor Bilharz Research Institute Kornaish El Nile, Warrak El-Hadar, Imbaba P.O. Box 30, Giza 12411, Egypt; 3 Department of Genomic Medicine, Cairo University Hospitals, Cairo University, Cairo 11566, Egypt; aidakelany@gmail.com; 4 MEU Research Unit, Middle East University, Amman 11831, Jordan; 5 Department of Medical Laboratories, College of Applied Medical Sciences, Qassim University, Buraydah 51452, Saudi Arabia; k.allemailem@qu.edu.sa; 6 Department of Pharmaceutics, College of Pharmacy, King Saud University, Riyadh 11451, Saudi Arabia; sbukhari@ksu.edu.sa; 7 Microbial Chemistry Department, National Research Centre, 33 El-Buhouth Street, Dokki, Giza 12622, Egypt

**Keywords:** iron oxide nanoparticles, *Streptomyces* sp. SMGL39, antibacterial, antibiofilm, XRD, FTIR, docking

## Abstract

One of the major global health threats in the present era is antibiotic resistance. Biosynthesized iron oxide nanoparticles (FeNPs) can combat microbial infections and can be synthesized without harmful chemicals. In the present investigation, 16S rRNA gene sequencing was used to discover *Streptomyces* sp. SMGL39, an actinomycete isolate utilized to reduce ferrous sulfate heptahydrate (FeSO_4_.7H_2_O) to biosynthesize FeNPs, which were then characterized using UV–Vis, XRD, FTIR, and TEM analyses. Furthermore, in our current study, the biosynthesized FeNPs were tested for antimicrobial and antibiofilm characteristics against different Gram-negative, Gram-positive, and fungal strains. Additionally, our work examines the biosynthesized FeNPs’ molecular docking and binding affinity to key enzymes, which contributed to bacterial infection cooperation via quorum sensing (QS) processes. A bright yellow to dark brown color shift indicated the production of FeNPs, which have polydispersed forms with particle sizes ranging from 80 to 180 nm and UV absorbance ranging from 220 to 280 nm. Biosynthesized FeNPs from actinobacteria significantly reduced the microbial growth of *Fusarium oxysporum* and *L. monocytogenes*, while they showed weak antimicrobial activity against *P. aeruginosa* and no activity against *E. coli*, *MRSA*, or *Aspergillus niger*. On the other hand, biosynthesized FeNPs showed strong antibiofilm activity against *P. aeruginosa* while showing mild and weak activity against *B. subtilis* and *E. coli,* respectively. The collaboration of biosynthesized FeNPs and key enzymes for bacterial infection exhibits hydrophobic and/or hydrogen bonding, according to this research. These results show that actinobacteria-biosynthesized FeNPs prevent biofilm development in bacteria.

## 1. Introduction

A rise in interest in the nanotechnology field has occurred recently because of the special and adjustable properties that nanoparticles display [1]. From these nanoparticles, iron oxide nanoparticles (FeNPs) have gathered significant interest [2] because of their numerous uses in medicine, environmental remediation, and catalysis [3,4]. Since the conventional methods of producing FeNPs use toxic substances and high temperatures, it has become important to explore eco-friendly substitutions [5].

Gram-positive bacteria actinomycetes, which have high contents of guanine and cytocine, have gained attention as a biological source for FeNPs synthesis [6]. These bacteria are dispersed to a large degree in diverse environments and have the ability to produce enzymes and bioactive substances [7]. These metabolic resources for the biosynthesis of FeNPs can be used as sustainable and biocompatible substitutions. FeNPs are biosynthesized through reduction in iron ions, and the produced nanoparticles are stabilized with the help of definite biomolecules that are produced by actinomycetes. Because of the special physicochemical properties of iron oxide nanoparticles (FeNPs), they have been employed in many different fields. In the field of medicine, FeNPs have been used as contrast agents in magnetic resonance imaging (MRI), enhancing the visibility of tissues and organs [3]. Due to their superparamagnetic characteristics, FeNPs can be applied in targeted drug delivery systems that target medicines to be released at precise locations [8]. The biocompatibility of FeNPs makes them viable options for a variety of biomedical applications, such as magnetic bioseparation, biosensing, and hyperthermia therapy [9].

Since antibiotic-resistant strains are becoming one of the greatest dangers to public health in the world, it is very important to explore new compounds with antibacterial properties [10]. The main problem with employing nanoparticles with antibacterial potentials is their significant toxicity, as observed in ZnO, for example [11,12]. Therefore, the biocompatibility and biological activity of antimicrobial nanoparticles are essential [13,14]. FeNPs are becoming an option in this field due to their bactericidal properties, as well as their in vivo and in vitro biocompatibility. Also, FeNPs have been demonstrated in mammalian cell cultures to be safe and nontoxic [15,16], a highly coveted property to employ in biomedical, clinical, and pharmaceutical applications. Fe_2_O_3_, magnetite Fe_3_O_4_, and limonite Fe_2_O_3·_H_2_O are the most significant FeNPs with biological activity [17,18].

FeNPs are a good option to fight microbial infections because of their vast surface area and tiny size, which improve their ability to interact with bacterial cells and increase their effectiveness to disrupt the bacterial membrane and induce oxidative stress [19]. Furthermore, FeNPs’ magnetic characteristics enable their precise and targeted distribution to certain infection sites. Iron oxide nanoparticles’ antibacterial properties offer strong potential for the creation of new tactics to fight bacterial infections, potentially offering a solution to the growing issues associated with antibiotic resistance in the healthcare field. Further investigations in this field could lead to the discovery of new approaches to treating infectious diseases more precisely and with fewer adverse effects [20].

Biofilms, which are populations of bacteria, adhere to either living or abiotic surfaces, and, as “protective clothing”, biofilms provide bacteria with tolerance to harsh conditions and can shield them from ultraviolet (UV) radiation, extreme pH, extreme temperature, high salinity, high pressure, malnutrition, antibiotics, and other risks [21]. Similar to humans, bacteria may interact with one another through a variety of biological pathways, which aid in their movement, development of pathogenicity, and production of biofilms. Such a system is supported by defense and virulence factor development signaling mechanisms [22]. This general technique of bacterial cell-to-cell communications is known as quorum sensing (QS), which is considered to be a difficult target for substances hostile to pathogenic agents.

The aim of this work was to biosynthesize the iron oxide nanoparticles from actinobacteria and to evaluate the antimicrobial activity and antibiofilm potential of the biosynthesized iron oxide nanoparticles. Also, this research aims to contribute to the growing body of knowledge in sustainable nanotechnology, with implications for both biomedical and industrial applications.

## 2. Results and Discussion

### 2.1. Marine Sample Collection and Isolation of Marine Actinobacteria

**Figure 1:**
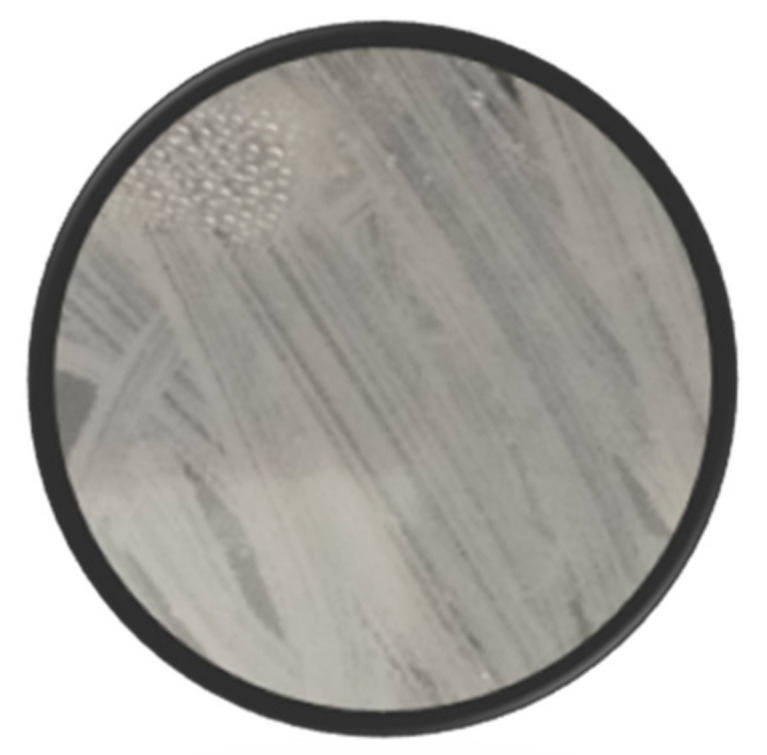
Morphological feature of isolated actinomycetes.

Two marine seawater samples were coded as MH1 and MH2 from Hurghada. Serial dilution and cultivation on appropriate media led to the isolation of five strains (LGO5, CH1, 46, 10, and 360). Differentiation of actinomycete colonies was based on their morphological feature; actinomycetes typically have unique morphology, appearing dense, raised, and often chalky or powdery in texture. They may also show a number of hues, such as white, gray, yellow, orange, or red, based on their morphology and color (Figure 1).

### 2.2. Preparation of Actinomycetes Filtrate and Screening Their Ability to Biosynthesis Iron Oxide Nanoparticles

**Figure 2:**
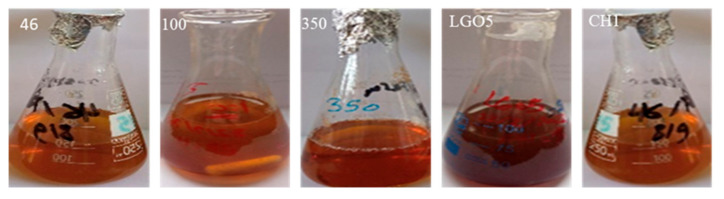
Screening of isolated actinomycetes’ (LGO5, CH1, 46, 10, and 360) abilities to biosynthesize iron oxide nanoparticles based on color change.

Five bacterial strains coded as LGO5, CH1, 46, 10, and 360 were cultured, and their filtrates were tested for the ability to biosynthesis iron oxide nanoparticles. The biosynthesis process was principally observed through a visible color change, where the color of the reaction mixture changed from a yellowish color to a dark brown, which indicates the formation of iron oxide nanoparticles, suggesting that specific strains have the potential to biosynthesize these nanoparticles efficiently. Further analysis and characterization of the produced nanoparticles will give useful insights into their compatibility for possible biotechnological applications (Figure 2).

### 2.3. Selection of a Potent Strain for Biosynthesis of Iron Oxide Nanoparticles

The strain exhibiting the greatest biosynthetic potential, as shown by the color transition from yellowish to dark brown, signifying the creation of iron oxide nanoparticles, was chosen for more investigation. Ultraviolet examination verified the existence of the plasmon band of the nanoparticles. Additional analysis of these nanoparticles will provide significant insights into their potential biotechnological uses.

### 2.4. Genetic Identification of the Most Potent Actinomycete Isolate

**Figure 3:**
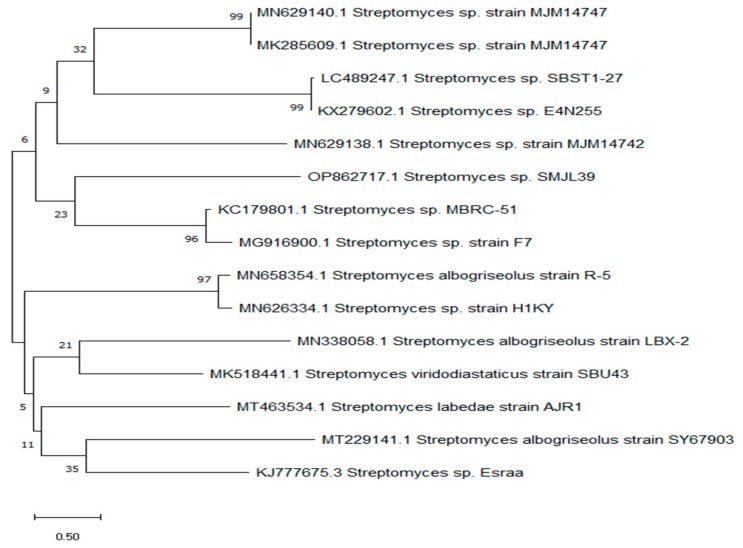
Phylogenetic relationship between *Streptomyces* sp. SMGL39 and other known sequences at the GenBank database.

Identification of the most potent bacterial strain was accomplished by sequencing the 16S rRNA gene. The DNA obtained from the isolate was isolated, identified, and compared to other known sequences in the GenBank database using the BLAST program. This was performed to calculate the similarity score and statistical significance of the matches. The BLAST online platform can be found at https://blast.ncbi.nlm.nih.gov/Blast.cgi accessed on 1 July 2023, and the analysis was conducted on 21 November 2022. The analysis showed that the 16S rRNA gene sequence of LGO5 had a similarity score of 99.64% with *Streptomyces* sp. Consequently, the sequence was submitted to the gene bank and assigned the accession number OP862717.1 as *Streptomyces* sp. SMGL39. The evolutionary history (Figure 3) was determined using the maximum likelihood method and the Tamura–Nei model [23]. The proportion of trees displaying the grouping of related taxa is shown next to the branches. The Tamura–Nei model was used to create a matrix of pairwise distances. The tree with the greatest log-likelihood value was selected as the initial tree for the heuristic search. The evolutionary study [24] was conducted using MEGA X. 

### 2.5. Biosynthesis of Iron Oxide Nanoparticles Using Streptomyces sp. SMGL39

**Figure 4:**
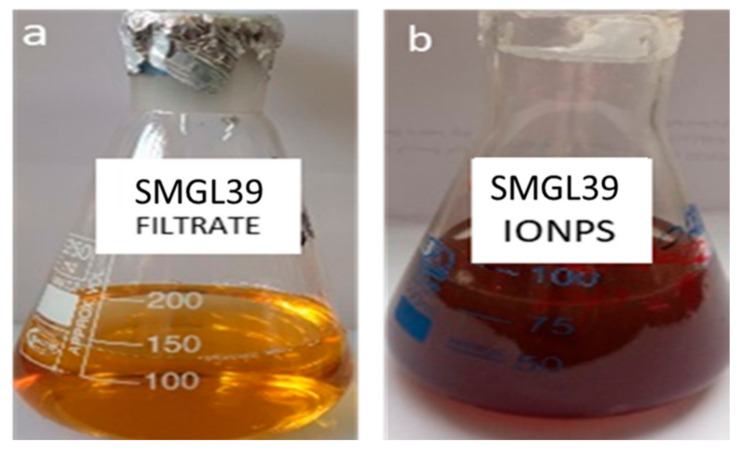
Color change of 3 mM ferrous sulfate from bright yellow into dark brown (using cell-free culture filtrate of actinobacteria: (**a**) culture filtrate, (**b**) culture filtrate with biosynthesized FeNPs).

The results in Figure 4 show that the synthesis of iron oxide nanoparticles was responsible for the dark brown color. The amount of nanoparticles produced per unit volume of bacterial solution was 80 micrograms/mL. Actinomycetes have the capacity to convert metal ions into their equivalent nanoscale metal particles. Full characterization of the biosynthesized iron oxide nanoparticles was also measured (Figure 4).

### 2.6. Characterization of Biosynthesized FeNPs

#### 2.6.1. Ultraviolet–Visible (UV–Vis) Spectral Analysis

**Figure 5:**
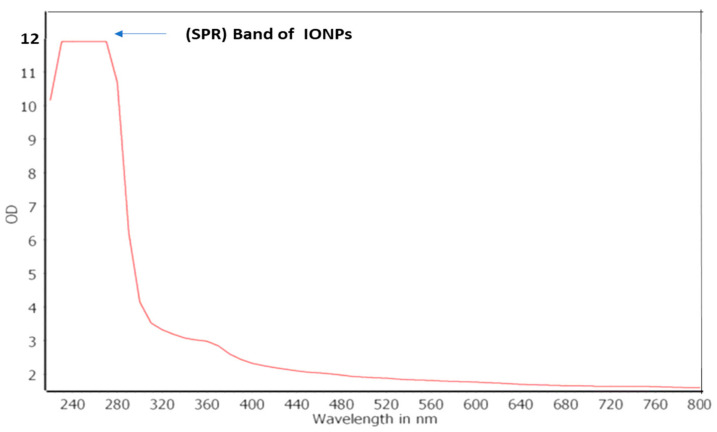
The UV–Vis spectra of the biosynthesized iron oxide nanoparticles (FeNPs) using cell-free culture filtrate of Actinobacteria SMGL39.

The spectra recorded from the FeNPs solution showed an absorbance peak at 220–280 nm, which is specific for the iron oxide nanoparticles (Figure 5). 

#### 2.6.2. Transmission Electron Microscopy (TEM) Analysis

**Figure 6:**
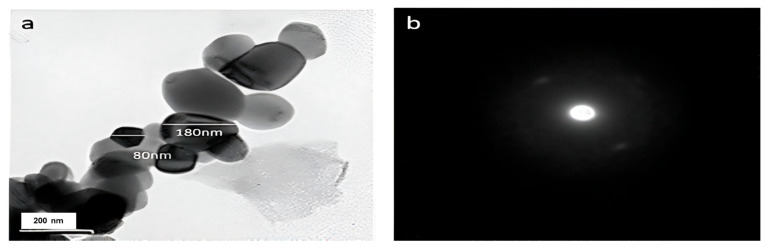
(**a**) TEM micrograph and (**b**) SAED images for the biosynthesized iron oxide nanoparticles (FeNPs) using cell-free culture filtrate of Actinobacteria SMGL39.

The transmission electron microscopy (TEM) analysis of the biosynthesized iron oxide nanoparticles (FeNPs) revealed polydisperse morphology, exhibiting a range of sizes between 80 and 180 nanometers (Figure 6a,b). The micrograph provided detailed insights into the size distribution and structural characteristics of the FeNPs, demonstrating the variety of particle forms in this size range. The polydispersed forms of the produced nanoparticles indicate the heterogeneous production process, highlighting the need to understand the biosynthetic processes that produce iron oxide nanoparticles with various sizes and shapes. The detailed TEM analysis significantly enhances a general understanding of the biosynthesis of FeNPs and their potential uses in different fields.

#### 2.6.3. X-ray Diffraction (XRD) Analysis

**Figure 7:**
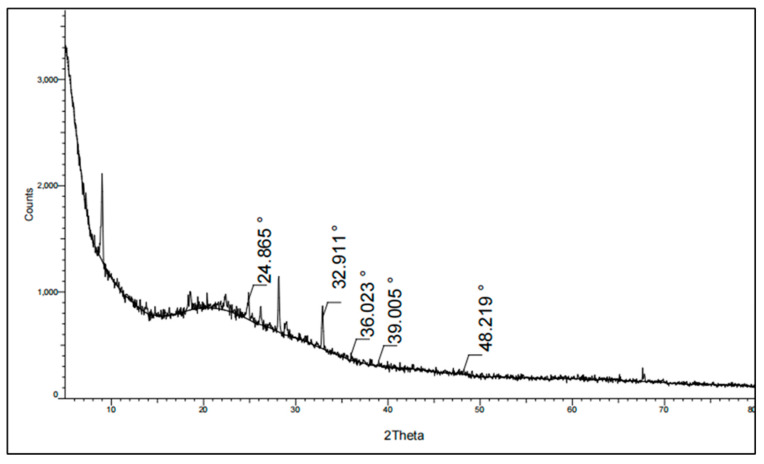
XRD pattern of biosynthesized iron oxide nanoparticles (FeNPs) using cell-free culture filtrate of Actinobacteria SMGL39.

X-ray diffraction (XRD) is considered one of the most important characterization techniques to study the structural and morphological properties of nanomaterials. Herein, the biosynthesized nanoparticles were examined via the XRD diffraction pattern, as shown in Figure 7. The XRD pattern of the biosynthesized FeNPs was detected at 24.8, 32.9, 36.02, 39.00, and 48.21 which is strongly similar to previously reported nanoparticles. 

#### 2.6.4. Fourier Transform Infra-Red (FTIR) Spectroscopic Analysis

**Figure 8:**
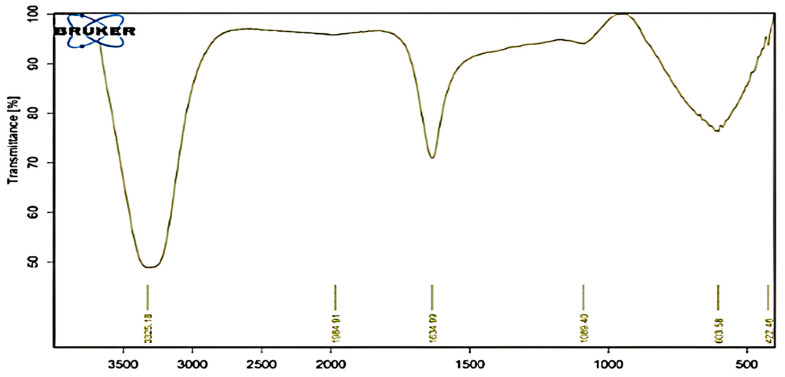
FTIR pattern of biosynthesized iron oxide nanoparticles (FeNPs) using cell-free culture filtrate of Actinobacteria SMGL39.

A peak at 3325 cm^−1^ in the FeNPs FTIR spectra is indicative of the stretching vibrations of the O-H group that are linked to isourea. The C=O group, which is linked to the CONH_2_ group, is responsible for the prominent peak at 1634 cm^−1^. The stretching vibration of the C-N group, which is attributed to aliphatic amines, is represented by the peak at 1089 cm^−1^. The characteristic peak of the carbonyl group of esters is located at 1985 cm^−1^ (Figure 8).

### 2.7. Antimicrobial Activity of Biosynthesized FeNPs 

**Figure 9:**
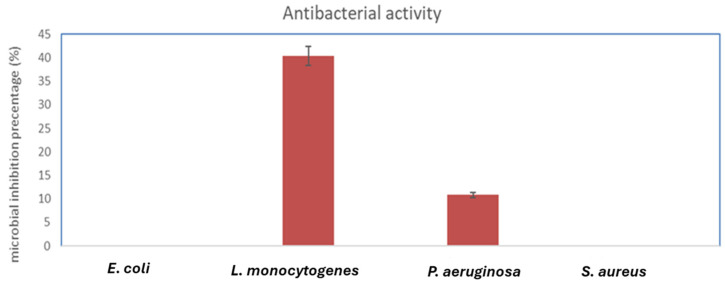
Antibacterial activity of FeNPs.

**Figure 10:**
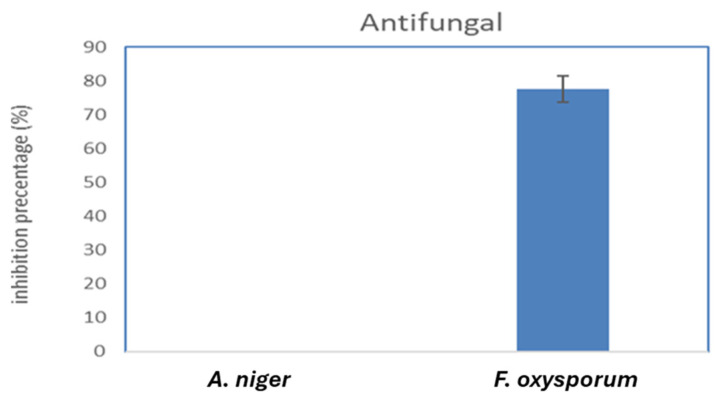
Antifungal activity of FeNPs.

Iron oxide nanoparticles significantly reduced the growth of *Fusarium oxysporum* microorganisms by 77.75%. This finding implies the effectiveness of the nanoparticles against this specific fungus. Similarly, iron oxide nanoparticles demonstrated moderate inhibitory activity against *L. monocytogenes* (ATCC 7644), resulting in a significant reduction in microbial growth by 40.42%. This indicates that these nanoparticles could be an effective choice for fighting the pathogenic bacteria *L. monocytogenes*, as they may have a bacteriostatic effect on it. Another bacterium that iron oxide nanoparticles successfully targeted was *P. aeruginosa* (ATCC 90902), which led to a 10.84% decrease in microbial growth (Figure 9). This demonstrates the capacity of the nanoparticles to prevent *P. aeruginosa* from growing, which is noted for its resistance to numerous traditional antibiotics. However, FeNPs showed no growth inhibition against *E. coli* (ATCC 8739), *MRSA*, and *Aspergillus niger*, highlighting their limited antibacterial effectiveness against these bacterial and fungal strains (Figure 9 and Figure 10).

Similar patterns were observed in the study of Azam et al., as their investigation revealed that iron oxide nanoparticles had no activity against *E. coli* and showed mild antibacterial activity toward *P. aeruginosa* [25]. Also, FeNPs have no antifungal action against *Aspergillus niger*, according to a Devi et al. investigation [26], which concurs with our findings. 

On the contrary, according to the study by Abdeen et al., the biosynthesized iron nanoparticles produced by *Fusarium oxysporum* exhibited antimicrobial activity against *E. coli* and *S. aureus* sp. [27]. Further, FeNPs synthesized by the *Musa ornate* flower sheath have antibacterial activity against *S. aureus* [28]. Also, iron oxide nanoparticles exhibited mild antibacterial activity against *S. aureus,* according to the investigation by Azam et al. [25].

Moreover, Batool and associates assessed the antibacterial efficacy of the green synthesized iron nanoparticles by *Phoenix dactylifera* extract against a variety of bacterial species and determined the maximal zone of inhibition against *E. coli* [29]. Research remains in progress to determine the mechanism of action with regard to the controversy related to the antibacterial characteristics of FeNPs. Several studies have produced contradictory results, with some demonstrating efficacy against *E. coli* and others showing no activity against this pathogen [30,31]. According to a study by Mohamed et al. (2015), iron nanoparticles cause oxidative damage to the bacterial cell wall; they exhibit antibacterial action against both Gram-positive and Gram-negative bacteria tested in their work. *Bacillus subtilis* is more susceptible to the antimicrobial effects of iron nanoparticles than *E. coli* and *S. aureus* [32]. The MIC of the biosynthesized iron oxide nanoparticles was measured toward the most susceptible pathogenic microbes, *L. monocytogenes* with MIC (125 µg/mL) and *Fusarium oxysporum* (62.25 µg/mL). 

### 2.8. Antibiofilm Activity of Biosynthesized FeNPs 

**Figure 11:**
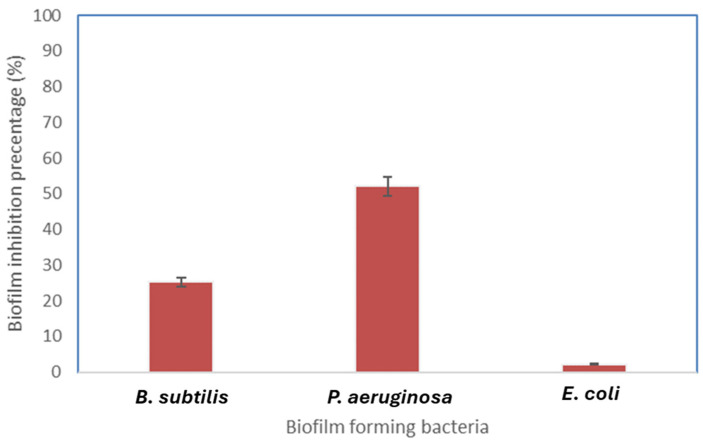
FeNPs’ inhibition of biofilm formation against *B. subtilis*, *P. aeruginosa*, and *E. coli*.

The FeNPs have strong antibiofilm action against *P. aeruginosa*, as demonstrated by the biofilm inhibition activity. However, there was no evidence of any inhibitory effect toward *E. coli* and rather mild activity against *Bacillus* biofilm formation (Figure 11).

By studying these findings from antimicrobial and antibiofilm results, we noticed that both the microbial and the biofilm formation activity of the Gram-negative bacteria *E. coli* were not affected by the biosynthesized FeNPs from actinobacteria. On the other hand, we noted that the biosynthesized FeNPs had strong antibiofilm activity against *P. aeruginosa* while mildly reducing its microbial growth, which can be resolved by the mechanism of action of the biosynthesized FeNPs that follow toward *P. aeruginosa*. These outcomes show the role of the biosynthesized FeNPs from actinobacteria in the inhibition of microbial growth and biofilm formation.

### 2.9. In Silico Studies

Molecular Docking Analysis 

**Figure 12:**
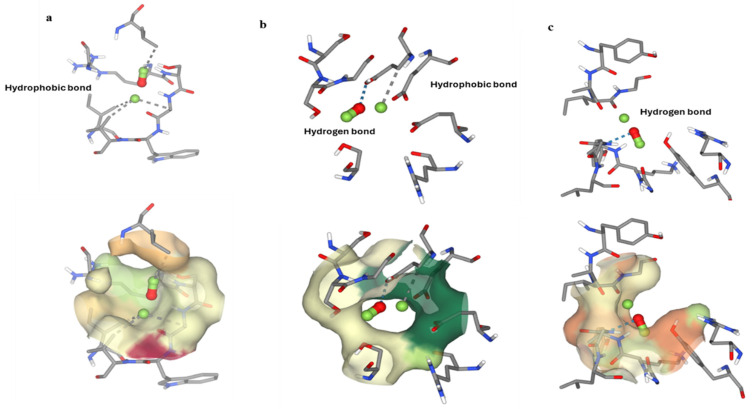
Molecular docking of FeNPs with enzymes essential for organizing bacterial infections via quorum sensing (QS) mechanisms: (**a**) Vfr, (**b**) LasR, and (**c**) QscR.

The biofilm inhibition activity demonstrated the FeNPs’ strong antibiofilm activity against *P. aeruginosa.* The distribution of quorum sensing (QS) by binding with significant important proteins such as Vfr, LasR, and QscR is one potential mechanism by which FeNPs inhibit biofilms. We investigated the molecular docking of iron oxide nanoparticles, with particular emphasis on the interactions between iron and important enzymes that are essential for coordinating bacterial infections via quorum sensing (QS) mechanisms. We specifically looked into its affinity for binding to enzymes like Vfr, LasR, and QscR. We explored a considerable hydrophobic interaction between iron metal and Vfr at definite amino acid residues, like ILE 32, LEU 75, ALA 89, and VAL 91; a binding energy of −2.5 resulted from this interaction (Figure 12a). The results showed the presence of hydrogen bonds and hydrophobic interactions between LasR and the amino acid GLU18 with a binding energy of −2.8 (Figure 12b). Further, we explored a powerful hydrogen bond interaction between QscR and VAL131, resulting in a binding energy of −2.8 (Figure 12c), while known inhibitors for the three proteins range from −6.0 to −9.0. These results reveal how well iron oxide nanoparticles attach to fundamental enzymes included in the pathophysiology of bacteria, highlighting the importance of using them in therapeutic interventions that aim to interfere with bacterial communication and infection processes. Iron oxide nanoparticles (FeNPs) have promising antibacterial activity as they can denature and inhibit enzyme activity [33]. In this regard, FeNPs can attach directly to enzymes, causing changes in their three-dimensional structure and, hence, their functioning [34]. When the nanoparticles attach to the enzyme’s active sites or other vital areas, they inhibit the substrate binding and, hence, the catalytic activity [35]. Additionally, the interactions between FeNPs and enzymes may result in conformational changes in the enzyme structure. These changes have the potential to inactivate the enzyme or significantly reduce its activity [36]. Because FeNPs bind to the active sites of certain enzymes, preventing the substrate molecules from accessing these sites, they may serve as competitive inhibitors for particular enzymes, reducing their activity [37]. As a type of noncompetitive inhibition, FeNPs may attach to sites on the enzyme other than the active site, changing the structure of the enzyme and reducing its activity [38].

## 3. Materials and Methods

### 3.1. Collection of Marine Samples and Isolation of Marine Actinobacteria 

Two marine water samples were collected from Hurghada (27.2579° N, 33.8116° E), Egypt, during September 2021 and coded as MH1 and MH2, then carefully transported to the laboratory under appropriate lighting and temperature conditions to protect their integrity. The samples were properly mixed in the lab to guarantee homogeneity and avoid bigger particles settling. Then, using sterile seawater or distilled water, serial dilutions ranging from 10^−1^ to 10^−6^ were made and then plated onto selective media, usually starch-casein agar, in order to isolate five different strains of actinomycetes, coded as LGO5, CH1, 46, 10, and 360. Actinobacteria grow slowly; thus, these agar plates were then incubated for 2 to 6 days at a temperature that is ideal for their growth (typically 25–30 °C). The colonies were selected manually based on colony morphology and color, then streaked onto new selective media to isolate pure cultures, and stored for extended periods of time at −80 °C. For greater accessibility and conservation, duplicate isolates were also submitted to specialized culture collections. The colonies that showed characteristic features for actinomycetes were selected for further analysis. Subculturing and purification: In order to reduce the risk of contamination and facilitate the identification of accurate strains, it is necessary to ensure that each colony begins with a single actinomycete cell by removing the chosen colonies and spreading them onto fresh agar plates using a sterile loop or needle to subcultivate them into pure cultures. 

### 3.2. Actinomycete Cell-Free Extract Production

A loopful of the isolated actinomyces cells were placed into a sterile tube containing 50 mL of ISP2 broth or another appropriate liquid growth medium at pH 7.2 with certain compositions (g L^−1^): yeast extract, 4.0; malt extract, 10.0; and dextrose, 4.0. To allow for adequate growth and metabolite production, the culture was incubated for six days at a temperature that is ideal for their growth (typically 25–30 °C) on a shaker incubator set at 150 to 200 rpm. Following incubation, the culture was centrifuged for 15 min at 10,000 rpm in order to separate the cells from the supernatant. The cell-free supernatant was carefully collected and filtered through a 0.22 μm membrane filter to exclude any remaining cells or debris [39].

### 3.3. Screening the Ability of Isolated Actinobacteria to Biosynthesize Iron Oxide Nanoparticles

Screening of the isolated bacterial strain ability to biosynthesize iron oxide nanoparticles was carried out according to Mohamed et al., with slight a modification of their procedure. A solution of 3 mM ferrous sulfate was prepared by dissolving 0.417 g of FeSO_4_.7H_2_O powder in 500 mL of distilled water. The cell-free extracts of the five actinomycetes strains were mixed with ferrous sulfate solution in a mass ratio of 2:5, respectively. The pH should then be adjusted to 11, which is the suitable pH for actinomycetes to produce iron oxide nanoparticles. After that, the mixtures of ferrous sulfate and actinomycetes extracts were then incubated for 48 h at an appropriate temperature. Solutions underwent a color shift from bright yellow to dark brown, signifying the efficient production of iron oxide nanoparticles from actinomycetes [32].

### 3.4. Genetic Identification of the Most Potent Bacterial Strain

The most potent bacterial strain that was able to biosynthesize iron oxide nanoparticles was cultivated for three days at 25 °C in starch casein broth media before being genetically identified by sequencing of its 16S rRNA gene. DNeasy Blood & Tissue Kit was used to extract the bacterial genomic DNA according to manufacturer instructions. Two universal primers (27F 5′-AGAGTTTGATCCTGGCTCAG-3’ and 1492R 5′-GGTTACCTTGTTACGACTT-3′) were used to undergo PCR amplification reactions. The final volume of the PCR amplification mixture was 50 μL (5 μL of 10 × Dream Taq Green PCR buffer, 2 μL of each 10 μmol dm−3 primers, 5 μL of 2 mmol dm−3 dNTP, 0.3 μL Taq DNA polymerase, and 0.5 μL of template DNA). The PCR reaction profile followed these conditions: 94 °C for 45 s, 55 °C for 60 s, and 72 °C for 60 s, then sequencing of the purified PCR products was carried out in Macrogen Company, South Korea. By comparing the resulted sequences with the similar known sequences accessible in the NCBI database using online BLAST alignment search tools (https://blast.ncbi.nlm.nih.gov/Blast.cgi) (accessed on 1 July 2023), we can resolve the homology and similarity of the 16S rRNA sequences and establish the phylogenetic trees by MEGA-11 software [40,41].

### 3.5. Characterization of Biosynthesized Iron Oxide Nanoparticles

#### 3.5.1. Ultraviolet–Visible (UV–Vis) Spectral Analysis

The process of converting ferrous ions into ferrous oxide nanoparticles was observed using spectrophotometry. This was performed by taking samples of the mixture at different time intervals and analyzing their UV–Vis spectra using a Jas-co-V-570 UV–visible spectrophotometer. The spectrophotometer was equipped with double-beam, 10 mm light path cells for measuring absorbance. The acquired spectra functioned as indicators for the formation of iron oxide nanoparticles. 

#### 3.5.2. Transmission Electron Microscopy (TEM) Analysis

The size and shape of the FeNPs generated at Nano Tech Egypt Centre were assessed using transmission electron microscopy (TEM). In order to prepare the sample, a volume of 2–4 µL of the sample solution was applied onto carbon-coated copper grids. A Philips 10 Techni electron microscope, operating at a wavelength (λ) of 0.0251 and an accelerating voltage of around 180 keV, was used to see the thin films after they had undergone air drying.

#### 3.5.3. X-ray Diffraction (XRD) Analysis

The X-ray diffraction (XRD) patterns of the generated FeNPs were measured using a PAN analytical X‘pert PRO X-ray diffractometer. The X-ray machine used in this study was manufactured by Philips in Eindhoven, Netherlands. It used Cu Ka1 radiation and operated at an approximate voltage of 40 kV and tube current of 30 mA. After applying the material to a glass substrate using the drop-coating method, the X-ray diffraction patterns were acquired by scanning at a range of 2θ from 10° to 80° with a scanning speed of 0.02°/min.

#### 3.5.4. Fourier Transform Infrared (FTIR) Spectroscopic Analysis 

The spectra of the generated FeNPs were acquired using the Broker vertex 80 v instrument. The spectra were collected in the region of 4000–400 cm^−1^ with a resolution of 4 cm^−1^ [42].

### 3.6. Assessment of Biological Activities

#### 3.6.1. Antimicrobial Assay

Determination of the antimicrobial activity of the prepared biosynthesized FeNPs was evaluated toward standard strains that represent pathogenic bacteria of Gram-negative bacteria including *Escherichia coli* (ATCC 8739), *P. aeruginosa* (ATCC 90902), Gram-positive bacterial isolates including MRSA and *L. monocytogenes* (ATCC 7644), and fungal strains such as *Fusarium oxysporium* and *Aspergillus niger*. According to earlier reports, the tests were conducted on 96 well-flat polystyrene plates. Initially, 150 μL of lysogeny broth was mixed with 10 μL of FeNPs. Next, 10 μL of log phase bacterial suspension (approximately 10^5^ viable cells per mL) was added. Finally, all inoculation plates were incubated for a whole night at 37 °C. Following incubation, clearance in the wells indicated the tested compounds’ positive effects, while the growth medium in the wells of the compounds that had no effect on the bacteria appeared opaque. After 20 h, the absorbance was measured in a Spectrostar Nano Microplate Reader (BMG LABTECH GmbH, Allmendgrun, Germany) at OD600. Growth medium plus distilled water served as the negative control, while pathogenic bacteria plus distilled water served as the positive control [43]. The MIC was also measured using the same method at different concentrations. 

#### 3.6.2. Anti-Biofilm Assay

The efficiency of the biosynthesized FeNPs in inhibiting biofilm formation in three standard strains that represent pathogenic bacteria, including Gram-positive bacteria (B. subtilis) and Gram-negative bacteria (*P. aeruginosa* and *E. coli*), was investigated using 96-well flat polystyrene plates. In summary, each well was filled with 180 µL of lysogeny broth (LB broth), then inoculated with 10 µL of pathogenic bacteria, followed by 10 µL of samples and control (final concentration of 500 µg mL^−1^) (excluding the test sample). The plates were placed in an incubator at a temperature of 37 °C for a duration of 24 hours. After this, the liquid in the wells was removed and rinsed with 200 µL of phosphate buffer saline (PBS) with a pH value of 7.2. This was performed to remove any bacteria that were not attached to the surface. The plates were then dried in a sterilized laminar flow for a period of 1 hour. To perform staining, 200 µL of crystal violet solution with a concentration of 0.1 percent weight/volume was added to each well and left for 1 h. After that, the surplus stain was removed, and the plates were allowed to dry. The dried plates were cleansed using 95% ethanol, and the optical density was then determined at a wavelength of 570 nm using a Spectrostar Nano Microplate Reader. The company BMG LABTECH GmbH is located in Allmendgrun, Germany [44].

### 3.7. In Silico Studies

#### Molecular Docking Studies

The quorum sensing and biofilm formation mechanisms in *P. aeruginosa* depend critically on LasR, Vfr, and QscR. The PDB database provided the structures of these three proteins: QscR (3SZT), Vfr (2OZ6), and LasR (4NG2). Following that, using the receptor–ligand interaction mode and a cutoff value of RMSD < 4.0 Å, the protein structures and the iron oxide molecule were loaded into the software (MOE, 2015.10). The top-scoring solution was selected for additional examination. In order to determine the molecular interactions between the proteins and the iron oxide molecule, we concentrated on examining the amino acid residues within a 4 Å radius of the proteins.

## 4. Conclusions

From our present investigation, we conclude that the biosynthesis of iron oxide nanoparticles from actinomycetes is an eco-friendly, inexpensive, and efficient method as it can be produced without any toxic chemicals. A bright yellow to dark brown color shift indicated FeNPs’ production, which was then characterized using UV, TEM, XRD, and FTIR analyses. The spectra recorded from the FeNPs solution showed an absorbance peak at 220–280 nm, which is specific for the iron oxide nanoparticles. TEM images showed the synthesis of polydispersed biogenic FeNPs with particle sizes from 80 to 180 nm. XRD analysis showed the crystalline properties of FeNPs. Also, we found that the biosynthesized iron oxide nanoparticles inhibited the growth of *Fusarium oxysporium* and *L. monocytogenes* (ATCC 7644), which are well known for their resistance to a variety of traditional antibiotics by 77.75%. and 40.42%, respectively. This indicates that these nanoparticles could be an effective choice for fighting these pathogens. However, FeNPs displayed mild antimicrobial activity against *P. aeruginosa* (ATCC 90902), shown by a 10.84% decrease in its microbial growth. Also, FeNPs showed no antimicrobial activity against *E. coli* (ATCC 8739), *MRSA*, and *Aspergillus niger*. Furthermore, the results showed that the FeNPs had strong antibiofilm action against *P. aeruginosa* and rather mild activity against *Bacillus* biofilm formation, but there was no evidence of any inhibitory effect on *E. coli*. During our investigation of the molecular docking of iron oxide nanoparticles, with particular emphasis on its affinity for binding to enzymes like Vfr, LasR, and QscR, we found a considerable hydrophobic interaction between iron metal and Vfr at definite amino acid residues, resulting in a binding energy of −2.5. A binding energy of −2.8 is due to the presence of a hydrogen bond and hydrophobic interactions between LasR and the amino acid GLU18. Further, we found a powerful hydrogen bond interaction between QscR and VAL131, resulting in a binding energy of −2.8, while known inhibitors for the three proteins range from −6.0 to −9.0. These results illustrate how well iron oxide nanoparticles bind to vital enzymes included in the pathophysiology of bacteria, leading us to recommend their potential use in therapeutic interventions to disrupt bacterial communication and infection mechanisms. These findings demonstrate the efficiency of the biosynthesized FeNPs from actinobacteria as antibacterial and biofilm-formation inhibitors.

## Data Availability

All data generated or analyzed during this study are included in this published article.
